# Effect of a high-dose bioactive components formula on reducing common diseases in term neonates delivered by cesarean section: a randomized, open-label, controlled trial

**DOI:** 10.3389/fnut.2026.1741431

**Published:** 2026-07-17

**Authors:** Ke Chen, Xi Zhang, Haixia Chen, Nianyang He, Yuqi Tang, Zhen Yang, Ziji Fang, Changqi Liu

**Affiliations:** 1 Department of Clinical Nutrition, Chengdu Women's & Children's Central Hospital, School of Medicine, University of Electronic Science and Technology of China, Chengdu, China; 2 Chengdu Women's & Children's Central Hospital, School of Medicine, University of Electronic Science and Technology of China, Chengdu, China; 3 Baoxing Central for Disease Prevention and Control, Sichuan, China; 4 Department of Child Health Care, Xindu Maternal and Child Health Care Hospital, Sichuan, China; 5 School of Exercise and Nutritional Sciences, San Diego State University, San Diego, CA, United States

**Keywords:** cesarean section, lactoferrin, milk fat globule membrane, nucleotide, probiotic

## Abstract

**Objective:**

To investigate the effects of a high-dose probiotic-lactoferrin (LF)-milk fat globule membrane (MFGM)-nucleotide combined formula on reducing the incidence risk of common diseases and related symptoms and improving growth and hemoglobin level in neonates born via cesarean section.

**Methods:**

This trial was a prospective, multicenter, open-label, randomized, controlled 3-month intervention study. A total of 150 eligible neonates were enrolled. All participants completed the full intervention and provided clinical data. All enrolled formula-feeding neonates were randomly assigned to either the intervention group (IG, *n* = 50) or the control group (CG, *n* = 50), and 50 exclusively breastfed neonates were assigned to the breastfeeding control group (BCG). The formula used in the IG contained 2 × 10^9^ CFU/100 g probiotics, 200 mg/100 g LF, added MFGM, and 55 mg/100 g nucleotides. In contrast, neonates in the CG were fed a formula containing 45–50 mg/100 g LF and 30–35 mg/100 g nucleotides, without probiotic or MFGM fortification. The primary outcome was the frequency of upper respiratory tract infections (URTIs). Secondary outcomes included the incidence of other respiratory, gastrointestinal, and allergic symptoms and diseases, as well as infant anthropometric indicators (including length, weight and head circumference) and the hemoglobin (Hb) concentration.

**Result:**

The incidence of URTIs over the 3-month intervention showed no significant difference among the three groups [the incidences of URTIs in CG, IG, and BCG: 22% (11/50), 18% (9/50), and 14% (7/50), respectively, χ^2^ = 1.084, p = 0.582] according to both intention-to-treat (TT) and per protocol (PP) analyses [incidence of URTIs in CG, IG, and BCG: 22.9% (11/48), 19.6% (9/46), and 14.9% (7/47), respectively, χ^2^ = 0.680, p = 0.712]. After the 3-month intervention, compared with infants in the CG, infants in the IG showed a reduced risk ration (RR) for functional dyspepsia [RR = 0.756, 95% confidence interval (95% CI) = 0.587–0.974] and eczema (RR = 0.647, 95% CI = 0.449–0.933). Hb levels in the CG and IG were both significantly higher than those in the BCG (107.35 ± 9.26 vs. 105.99 ± 7.01 vs. 101.42 ± 7.07 g/L for CG, IG, and BCG, respectively; *F* = 9.629, *p* < 0.001), with no statistically significant difference between the CG and IG. No significant differences in anthropometric indicators were observed among the three groups (*p* > 0.05).

**Conclusion:**

This study found no adverse effects associated with 3-month supplementation of a high-dose probiotic-LF-MFGM-nucleotide combined formula, supporting its safety for term neonates delivered by cesarean section. The primary outcome, URTI incidence, did not differ significantly among groups. Exploratory analyses suggested lower risks of functional dyspepsia and eczema in the intervention group; however, these findings should be considered preliminary and require confirmation in larger adequately powered studies. The intervention was well tolerated and showed safety comparable to standard feeding practices.

## 1 Background

In 2019, an estimated 5.3 million children under 5 years of age died worldwide, according to the World Health Organization (WHO) (1). The global number of under-5 deaths in 2019 was estimated at 5.30 million (95% UR: 4.92–5.68 million), and nearly half of these deaths (49.2%) were due to infectious diseases, with respiratory and gastrointestinal infections accounting for 13.9% and 9.1%, respectively. Meanwhile, over the past 30 years, allergic diseases have risen sharply, affecting approximately 433 million people globally (2) and becoming a major public health issue in most developed countries (3), including China (4). Preventing respiratory, gastrointestinal, and allergic diseases is therefore crucial for child health and development.

As is well known, vaginal delivery enables neonates to be exposed to their mothers' vaginal microbiota during the birthing process (5, 6). These microorganisms play a vital role in the establishment of the infant gut microbiota and its subsequent healthy development. In contrast, infants delivered via cesarean section (C-section) are deprived of this early microbial contact (7). Their gut microbiota may consist predominantly of bacteria from the hospital environment, which could potentially include pathogens. Additionally, C-section delivery may disrupt the balance of the infant gut microbiota, thus impeding normal immune system development and elevating the risk of certain diseases in infants and toddlers. However, the balance of the gut microbiota can be partially restored through several intervention strategies (8–11). These interventions may also enhance intestinal and respiratory immunity, thereby increasing the body's capacity to combat infections.

Breastfeeding for the prevention of infant disease is a cost-effective strategy (12, 13). The active components in breast milk significantly influence infant immunity and intestinal microflora. These components encompass beneficial bacteria, human milk oligosaccharides (HMO), immunoglobulins, lactoferrin (LF), lysozymes, milk fat globule membrane (MFGM), nucleotides, and others, which synergistically protect and support the infant immune system and gastrointestinal health (14). Beneficial bacteria have been shown to possess antibacterial and immunomodulatory properties, protecting neonates from infections (15). LF offers defense against Gram-negative enteropathogens by sequestering iron, which is crucial for bacterial growth (16). MFGM enhances the survival of probiotics in the gastrointestinal tract by binding to them through electrostatic interactions, thereby improving their adhesion capabilities (17). Nucleotide metabolism may also play an indirect role in regulating fecal flora (18, 19).

Although numerous infant formula products currently on the market have incorporated one or several of the above-mentioned nutrients with immunomodulatory effects to enhance the immune status of formula-fed infants, several issues remain. First, despite mimicking the composition of human milk by adding bioactive components with immunomodulatory and intestinal microecological regulatory properties, most commercially available products focus on only one or two components, and therefore may be unable to fully exert regulatory effects on infant immunity and intestinal microecology. Second, as previously stated, there are significant variations in the dosage of each nutrient added across different formula products (20), making it challenging to assess their precise clinical efficacy.

Breastfeeding appears to have a positive and dose-dependent impact on respiratory health, particularly during early childhood and in high-risk populations (21). Therefore, theoretically, higher doses of combined nutrients may be more beneficial for the health, growth, and development of high-risk infants. Nevertheless, there is a paucity of evidence-based data to support this assumption. Although cumulative evidence strongly supports the individual and synergistic functions of LF, MFGM, probiotics, and nucleotides in reducing RTIs risk, the current peer-reviewed literature lacks large-scale randomized controlled trials evaluating the high-dose combined effect of all four components – probiotics, MFGM, LF, and nucleotides – specifically in neonates delivered by C-section.

Accordingly, we postulated the following hypothesis: compared with lower-dose combinations, a higher-dose combination of probiotics, LF, MFGM, and nucleotides would be more beneficial in reducing the incidence of common diseases and promoting the growth and development of neonates born via C-section. To address this hypothesis, we conducted an intervention study to evaluate the effects of a first-stage infant formula containing higher doses of these combined nutrients on growth parameters and the incidence of allergic, respiratory, and gastrointestinal diseases in term neonates delivered by C-section.

## 2 Subjects and methods

### 2.1 Study design and participants

This study was a prospective, multicenter, open-label, randomized, controlled intervention study conducted from July 1, 2024 to August 31, 2025. Healthy neonates were recruited from three sites in Sichuan Province, China: Chengdu Women and Children's Center Hospital, Baoxing Center for Disease Control and Prevention, and Zhoujia Hospital of Hanyuan County.

#### 2.1.1 Inclusion criteria

(1) Full-term neonates born by C-section at 37–42 weeks of gestation, healthy, weighing ≥ 2,500 g and <4,000 g, whose parents voluntarily chose formula feeding immediately after birth, with no sex restrictions;

(2) Mothers of neorate without obstetric high-risk factors during pregnancy, such as gestational hypertension syndrome, eclampsia and preeclampsia, gestational diabetes, gestational cholestasis, or infectious diseases such as hepatitis B and HIV, as well as no history of alcohol or drug abuse;

(3) Parents or main guardians of neonates agreed to the collection of infant peripheral blood samples for hemoglobin testing during this study;

(4) Regular health care at local medical institutions and receipt of feeding guidance from child health care workers;

(5) Only the addition of probiotics for therapeutic purposes was permitted;

(6) Written informed consent signed by parents or main guardians.

#### 2.1.2 Exclusion criteria

(1) History of asphyxia or Neonatal Intensive Care Unit (NICU) hospitalization at birth;
(2) Congenital defects or anomalies;
(3) First-degree relatives of neonates with allergic diseases diagnosed by clinicians (including but not limited to eczema, asthma, allergic proctocolitis, allergic rhinitis, hay fever, and food allergies);
(4) Neonates with serious primary diseases of major organs or systems such as the heart, liver, kidney, or hematopoietic system;
(5) Use of experimental medications or participating in another clinical trial before screening;
(6) Known allergies to any components of the formula products used in the study;
(7) Other reasons deemed inappropriate for participation by the researchers, such as factors affecting efficacy evaluation or poor compliance.

#### 2.1.3 Withdrawal criteria

(1) Incorrect inclusion and misdiagnosis;
(2) Development of new allergies to formula product ingredients during the study;
(3) Inability to take formula orally during the intervention;
(4) Poor compliance due to changing formula for non-medical reasons;
(5) Occurrence of adverse events, abnormal laboratory test results, or other medical conditions related to the study formula;
(6) Withdrawal of informed consent by parents or main guardians during the intervention.

The enrollment and research plan were reviewed and approved by the Ethics Committee of Chengdu Women and Children's Central Hospital [Ethical Approval No.: Scientific Ethics Review 2024 (46)-2]. All parents/guardians were fully informed of the study details and provided written informed consent. This study was registered with the China Clinical Trial Center (registration number: ChiCTR2400089222; https://www.chictr.org.cn/showproj.html?proj=241320) and was supported by the China Medical Education Association (No. 2024-011). Project planning began on July 1, 2024, with official launch and registration on September 1, 2024. Participant recruitment started on October 1, 2024, and the intervention was completed on April 31, 2025.

### 2.2 Randomization and blinding

Randomization was conducted by a biostatistician not involved in trial implementation. Using the RAND function in Excel, random numbers were generated to assign eligible participants to either the intervention group (IG), fed a first-stage infant formula with higher doses of probiotics, LF, MFGM, and nucleotides, or the control group (CG), fed a formula with lower doses of these component. Participants who met the inclusion criteria were sequentially numbered and randomly allocated, resulting in 50 neonates per group. Laboratory personnel, the data manager, and the statistician were blinded to group assignments until completion of data analysis.

### 2.3 Intervention

All eligible neonates were randomly assigned to either the IG or the CG and received free first-stage infant formula that complied with the new “Food Safety National Standard Infant Formula” (GB10765-2021). The formula used in the IG contained 2 × 10^9^ CFU/100 g probiotics (a triple strain of *Bifidobacterium animalis* subsp. *lactis* BB-12, HN019, and Bi-07), 200 mg/100 g LF, added MFGM, and nucleotides (cytidine monophosphate, uridine monophosphate, adenosine monophosphate, inosine monophosphate, and guanosine monophosphate) at 55 mg/100 g. In contrast, neonates in the CG were fed a formula containing 45–50 mg/100 g LF and nucleotides (cytidine monophosphate, uridine monophosphate, adenosine monophosphate, and guanosine monophosphate) at 30–35 mg/100 g, without probiotic or MFGM fortification. The total intervention period was 3 months.

In addition, a control group of exclusive breastfed neonates (BCG) was included (*n* = 50). The inclusion and exclusion criteria for the BCG were the same as those for the formula-fed groups, except that feeding consisted of exclusive breastfeeding immediately after birth. Neonates in the BCG were matched 1:1 with those in the IG by sex at enrollment. Due to the limited number of births in the study area, a slight difference in sex composition between groups occurred. Nevertheless, this did not result in statistically significant inter-groups bias.

During the intervention period, infants attended monthly on-site follow-up visits. Parents and caregivers could consult the study team at any time regarding questions or concerns. Any illnesses during the trial were managed by pediatricians according to standard clinical guidelines.

### 2.4 Data collection

After enrollment, study staff performed assessments, recorded data in the clinical report form (CRF), and collected laboratory samples according to the protocol. Parents or primary caregivers completed a questionnaire administered by a trained on-site health worker, lasting approximately 30 min. The questionnaire collected demographic information (e.g., birth weight and sex), family socioeconomic information (e.g., parental education level, monthly family income, registered residence, and number of household residents). The primary outcome was the frequency of upper respiratory tract infections (URTIs) during the 3-month intervention period. Diagnosis, treatment, and clinical management of URTIs were determined according to the relevant guidelines of the Chinese Medical Association (22). Secondary outcomes included the incidence of other respiratory, gastrointestinal, and allergic symptoms and diseases, as well as infant anthropometric indicators (length, weight, and head circumference) and peripheral blood hemoglobin (Hb) concentration. Functional dyspepsia was defined according to the Rome IV criteria for pediatric functional gastrointestinal disorders. Clinicians used the CRF to record the duration, frequency, therapeutic drugs used, and related symptoms and signs of respiratory, gastrointestinal, and allergic diseases during the 3-month intervention.

### 2.5 Anthropometric measurements

Anthropometric examinations were conducted by the same trained health care workers (n = 6) at each hospital when infants were 1.5 and 3 months of age, using standardized techniques to minimize inter-examiner error. Duplicate measurements were performed for all infants. The inter-examiner coefficients of variation for weight, length, and head circumference (HC) were <5%. Weight was measured using a weighing scale (100 Med, Beijing, China) to the nearest 100 g, with infants wearing minimal clothing and bare feet. Length was measured in the supine position using a supine scale (Haode, Guangzhou, China) to the nearest 0.1 cm.

### 2.6 Blood sample collection and Hb assessment

Peripheral blood samples were collected from the infant heel at 1.5 and 3 months of age. Prior to collection, the site was massaged or warmed with a hot compress to enhance blood circulation. The skin was disinfected with 75% ethanol and allowed to dry. A disposable lancet was used to puncture the heel, with a puncture depth of <3 mm. The first drop of blood was discarded, and subsequent drops were collected into EDTA anticoagulant tubes. Blood samples were immediately stored at 4 °C to prevent microhemolysis and were analyzed for Hb concentration using the hemoglobin cyanide method (Maker, Chengdu, China) (23) within 4 h of collection. During transportation, samples were protected from shock, light exposure, and temperature fluctuations.

### 2.7 Compliance

Compliance with formula feeding was monitored using recording tables, in which parents or caregivers recorded the numbers of spoons consumed daily (spoon = 4.3 g). Distribution (performed by field workers) and consumption of the formula were closely supervised. Acceptability of the formula was assessed by a short questionnaire administered after feeding. Field workers visited each infant's home every weekend to examine and verify the quantity of formula consumed.

### 2.8 Sample size

Sample size estimation was based on a previous study (24) that evaluated the preventive effects of probiotics at a daily dose of 1 × 10^9^ CFU on URTI symptoms in children. In that study, the frequency of URTIs episodes during the third month of supplementation was 0.24 episodes/person in the intervention group [n = 38 for per-protocol (PP) analysis; n = 50 for intention-to-treat (ITT) analysis] compared with 0.73 episodes/person in the placebo group (n = 33 for PP analysis; n = 50 for ITT analysis), with a significant difference (*p* < 0.001). Based on these findings, 50 participants were enrolled in each group to ensure sufficient power for ITT analysis to detect a statistically significant difference in URTI incidence between groups.

### 2.9 Statistical analysis

Data distributions were assessed for normality using the *Kolmogorov-Smirnov* goodness-of-fit test. Normally distributed data are expressed as mean ± standard deviation, and skewed data as median (25th and 75th percentiles). All statistical tests were two-tailed, and *p* < 0.05 was considered statistically significant. The χ^2^ test was used for inter-group comparisons of categorical clinical and demographic variables. To compare intervention effects among groups, multiple comparisons of parameter changes after the intervention were performed using the *Student-Newman-Keuls* test for normally distributed and homogeneous data (growth parameters and Hb levels), or the *Kruskal-Wallis* rank test for skewed data (days with common symptoms during the intervention). Statistical analyses were conducted using IBM SPSS Statistics 29.0.2 for Mac.

## 3 Results

### 3.1 Baseline characteristics

A total of 150 eligible neonates were enrolled in the study. All participants completed the full intervention procedure and provided clinical and demographic data. All formula-fed neonates were randomly assigned to either the IG (*n* = 50) or the CG (*n* = 50), and 50 exclusive breastfed neonates were assigned to the BCG. There were no losses in follow-up, and all participants completed the CRF and were included in ITT analysis. However, nine infants were excluded from the PP analysis due to significant protocol deviations, among whom 4 cases in the IG and 2 cases in the CG were attributed to changing formula for non-medical reasons. The compliance rate was 25% in the intervention group and 30% in the control group, including 4 in the IG and 2 in the CG. The compliance rate was 92.0% (46/50) in the IG and 96.0% (48/50) in the CG. Consequently, the PP dataset included 141 infants: 46 in the IG, 48 in the CG, and 47 in the BCG.

**Figure 1:**
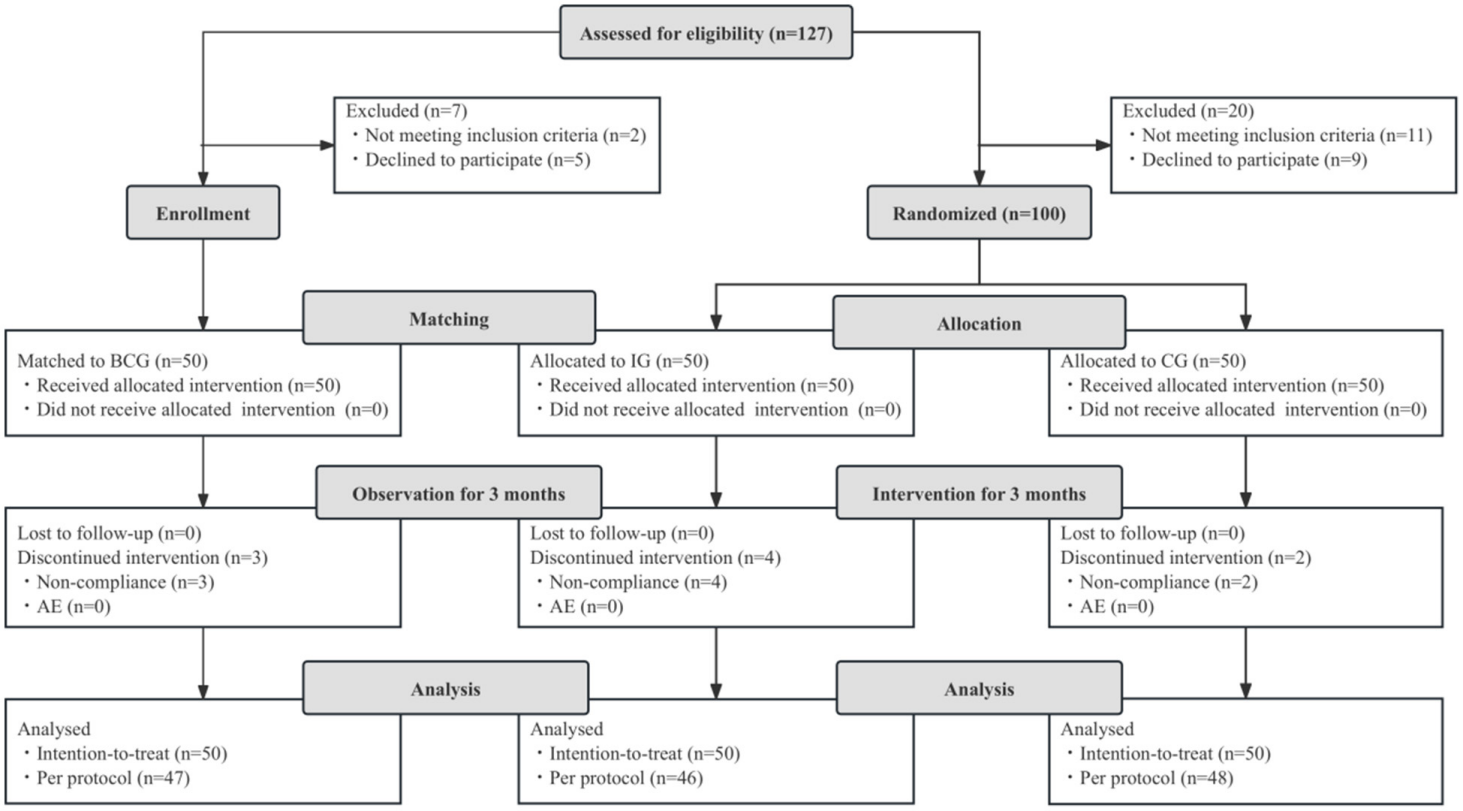
Flowchart of subject enrollment and study progress. IG, intervention group; CG, control group; BCG, breastfeeding control group (matched 1:1 with neonates in the IG by sex); AE, adverse events.

No adverse events related to the investigational product were reported throughout the study. Figure 1 shows the flowchart for participant recruitment, allocation, follow-up, and analysis. At baseline, there were no significant differences among the three groups in terms of sex distribution, birth weight, parental education level, household per capita income, registered residence, or numbers of household residents (*p* > 0.05, Table 1).

**Table 1:** Basic clinical and demographic information of the three groups [means ± SD].

Items	CG (*n* = 50)	IG (*n* = 50)	BCG (*n* = 50)	*F/χ^2^* values	*p* values
Sex [boy, *n* (%)]^*^	27 (54.0)	22 (44.0)	24 (48.0)	1.014	0.602
Birth weight (kg)^*^	3.162 ± 0.341	3.284 ± 0.453	3.230 ± 0.301	1.336	0.266
Parental education level ^a^*					
Junior high school and below	5	12	9	10.762	0.215
Senior High School/Technical Secondary School	12	7	10		
Junior College/Vocational College	32	26	30		
Bachelor's degree or above	1	5	1		
Household per capita income ^*^				11.410	0.216
<2,000 RMB¥	0	2	0		
2,001–3,000 RMB¥	19	14	21		
3,001–4,000 RMB¥	13	11	5		
4,001–5,000 RMB¥	6	7	9		
5,001–6,000 RMB¥	8	10	11		
>6,001 RMB¥	4	6	4		
Registered residence [urban, *n* (%)]^*^	45 (90.0)	39 (78.0)	41 (82.0)	6.592	0.159
Number of household residents [*n* < 4, *n* (%)]^*^	25 (50.0)	28 (56.0)	30 (60.0)	1.025	0.401

^a^, The highest parental education level was used for statistical analysis. ^*^, No significant differences among the three groups (*p* > 0.05); SD, standard deviation; IG, intervention group; CG, control group; BCG, breastfeeding control group (matched 1:1 with neonates in the IG by sex).

### 3.2 Effects of interventions on the primary outcome

The incidence of URTIs over the 3-month intervention did not differ significantly among the three groups [incidence in CG, IG, and BCG: 22% (11/50), 18% (9/50), and 14% (7/50), respectively; χ^2^ = 1.084, *p* = 0.582] according to the ITT analysis.

PP analyses yielded consistent results, with no significant difference among the three groups [incidence in CG, IG, and BCG: 22.9% (11/48), 19.6% (9/46), and 14.9% (7/47), respectively; χ^2^ = 0.680, *p* = 0.712]. These findings indicate that 3-month supplementation did not result in statistically significant reduction in URTI incidence, and the results were consistent across both ITT and PP analyses.

### 3.3 Effects of intervention on symptom duration of respiratory, gastrointestinal, and allergic diseases

During the 3-month study period, total occurrence days of common respiratory, gastrointestinal, and skin symptoms, as well as incident rate per 100 intervention days and inter-group comparisons, are presented in Table 2. Significant differences among the three groups were observed in total occurrence days of coughing, runny nose, loose stools, choking, retching/vomiting, stool with milk flaps, food residues, or sour odor, refusal to eat, and eczematous skin changes (all *p* values <0.05). *Kruskal-Wallis* nonparametric analysis indicated that, compared with infants in the CG, infants in the IG experienced significantly fewer total occurrence days of coughing, runny nose, retching/vomiting, stool with milk flaps, food residues, or sour odor, and refusal to eat during the intervention period (all *p* values <0.05). However, the occurrence of loose stools was significantly higher in the IG than in the CG (*p* < 0.05). When compared with infants in the BCG, no significant differences were observed in total occurrence days for any of the common symptoms (all *p* values > 0.05).

**Table 2:** Comparison of the duration of common symptoms in infants during the intervention period.

Common symptoms	CG (*N* = 50)		IG (*N* = 50)		BCG (*N* = 50)		*p* values
	Total occurrence days	Incident rate per 100 intervention days (%)†	Total occurrence days	Incident rate per 100 intervention days (%)†	Total occurrence days	Incident rate per 100 intervention days (%)†	
Cough^*^	135	3.000a	81	1.800b	68	1.511b	0.002
Runny nose^*^	165	3.667a	98	2.178b	110	2.444b	<0.001
Stuffy nose	291	6.467a	231	5.133a	228	5.067a	0.179
Fever	84	1.867a	74	1.644a	75	1.667a	0.683
Loose stools^$*^	201	4.467a	361	8.022b	372	8.267b	0.006
Increased frequency of bowel movements	258	5.733a	314	6.978ab	380	8.444b	0.053
Colic of the intestines	500	11.111a	356	7.911a	409	9.089a	0.083
Dry stool^&^	601	13.356a	465	10.333a	432	9.600a	0.114
Choking^*^	313	6.956a	217	4.822ab	195	4.333b	0.005
Increased belching/bloating/anal gas	528	11.733a	573	12.733a	468	10.400a	0.285
Retching/vomiting^*^	536	11.911a	273	6.067b	296	6.578b	<0.001
Stool with milk flaps/food residues/sour odor^*^	941	20.911a	621	13.800b	675	15.000b	0.001
Reflux	317	7.044a	342	7.600a	435	9.667a	0.077
Decreased appetite	261	5.800a	294	6.533a	304	6.756a	0.555
Dysphoria	362	8.044a	427	9.489a	465	10.333a	0.172
Refusal to eat^*^	190	4.222a	98	2.178b	85	1.889b	<0.001
Eczematous changes of the skin^*^	335	7.444a	223	4.956ab	113	2.511b	<0.001
Erythematous changes in the skin	157	3.489a	137	3.044a	185	4.111a	0.100
Skin wheal-like changes	8	0.178a	16	0.356a	6	0.133a	0.658

†, A total of 4,500 study days (calculation as 90 days × 50 infants per group). Symptom incidence per 100 intervention days = duration of a symptom/total intervention days × 100%. ^*^, *Kruskal-Wallis* test for multiple independent nonparametric samples; *p* < 0.05. $, Loose stools refer to Bristol stool score types 6–7. &, Dry stool refers to Bristol score types 1–3. IG, intervention group; CG, control group; BCG, breastfeeding control group (matched 1:1 with neonates in the IG by sex). The letters (a, b, c) indicate the results of multiple pairwise comparisons following the *Kruskal–Wallis* test for multiple independent nonparametric samples. Groups sharing the same letter are not significantly different from each other, whereas groups with different letters are considered to differ significantly (*P* < 0.05).

### 3.4 Effects of probiotic intervention on the morbidity of respiratory, gastrointestinal, and allergic diseases

**Figure 2:**
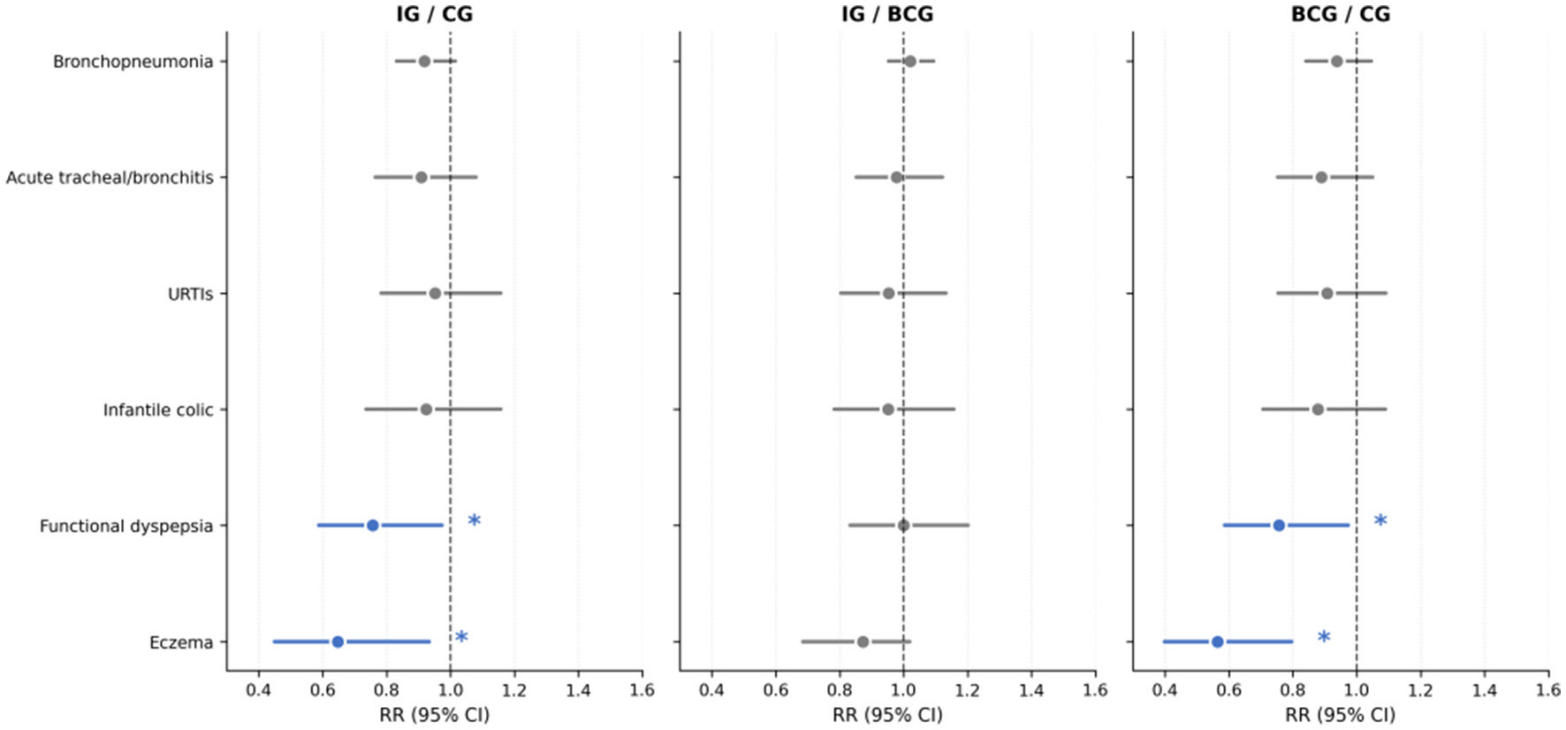
Effects of intervention on the risk ratios of common diseases during the study period. URTIs: upper respiratory tract infection; RR, risk ratio; 95% CI, 95% confidence interval for risk ratio; IG, intervention group; CG, control group; BCG, breastfeeding control group (matched 1:1 with neonates in IG by sex); *, significant lower of RR values (*p* < 0.05).

Morbidity data were collected over a median duration of 90 days (interquartile range: 82–101 days) for both groups. Table 3 summarized the incidences and the risk ratios (RR) for respiratory, gastrointestinal, and allergic diseases during the study period. Compared to the CG, the IG showed a lower RR for functional dyspepsia [RR = 0.756, 95% confidence interval (95% CI) = 0.587–0.974], with an absolute risk reduction (ARR) of 20% and a number needed to treat (NTT) of 5, as well as for eczema (RR = 0.647, 95% CI = 0.449–0.933), with an ARR of 24% and an NTT of 4. In contrast, the RRs for bronchopneumonia, acute tracheal/bronchitis, URTIs, infantile colic, functional dyspepsia, and eczema in infants in the IG were comparable to those observed in the BCG (*p* > 0.05, Figure 2).

**Table 3:** Morbidity of respiratory, gastrointestinal, and allergic diseases in infants during the study period.

Common diseases during study	Incidence (%)			RR values (95%CI)		
	CG (*N* = 50)	IG (*N* = 50)	BCG (*N* = 50)	IG/CG (*N* = 50)	IG/BCG (*N* = 50)	BCG/CG (*N* = 50)
Bronchopneumonia	10.0	2.0	6.0	0.918 (0.831, 1.015)	1.021 (0.953, 1.094)	0.938 (0.841, 1.045)
Acute tracheal/bronchitis	20.0	12.0	10.0	0.909 (0.765, 1.080)	0.978 (0.852, 1.122)	0.889 (0.753, 1.050)
URTIs^*^	22.0	18.0	14.0	0.951 (0.782, 1.158)	0.953 (0.803, 1.132)	0.907 (0.754, 1.091)
Infantile colic	28.0	22.0	18.0	0.923 (0.736, 1.158)	0.951 (0.782, 1.158)	0.878 (0.707, 1.090)
Functional dyspepsia	38.0	18.0	18.0	0.756 (0.587, 0.974)	1.000 (0.832, 1.202)	0.756 (0.587, 0.974)
Eczema	56.0	32.0	22.0	0.647 (0.449, 0.933)	0.872 (0.685, 1.019)	0.564 (0.399, 0.797)

^*^, URTIs: upper respiratory tract infection; RR, risk ratio; 95% CI, 95% confidence interval for the risk ratio; IG, intervention group; CG, control group; BCG, breastfeeding control group (matched 1:1 with neonates in the IG by sex).

### 3.5 Effects of intervention on growth parameters and Hb levels

**Figure 3:**
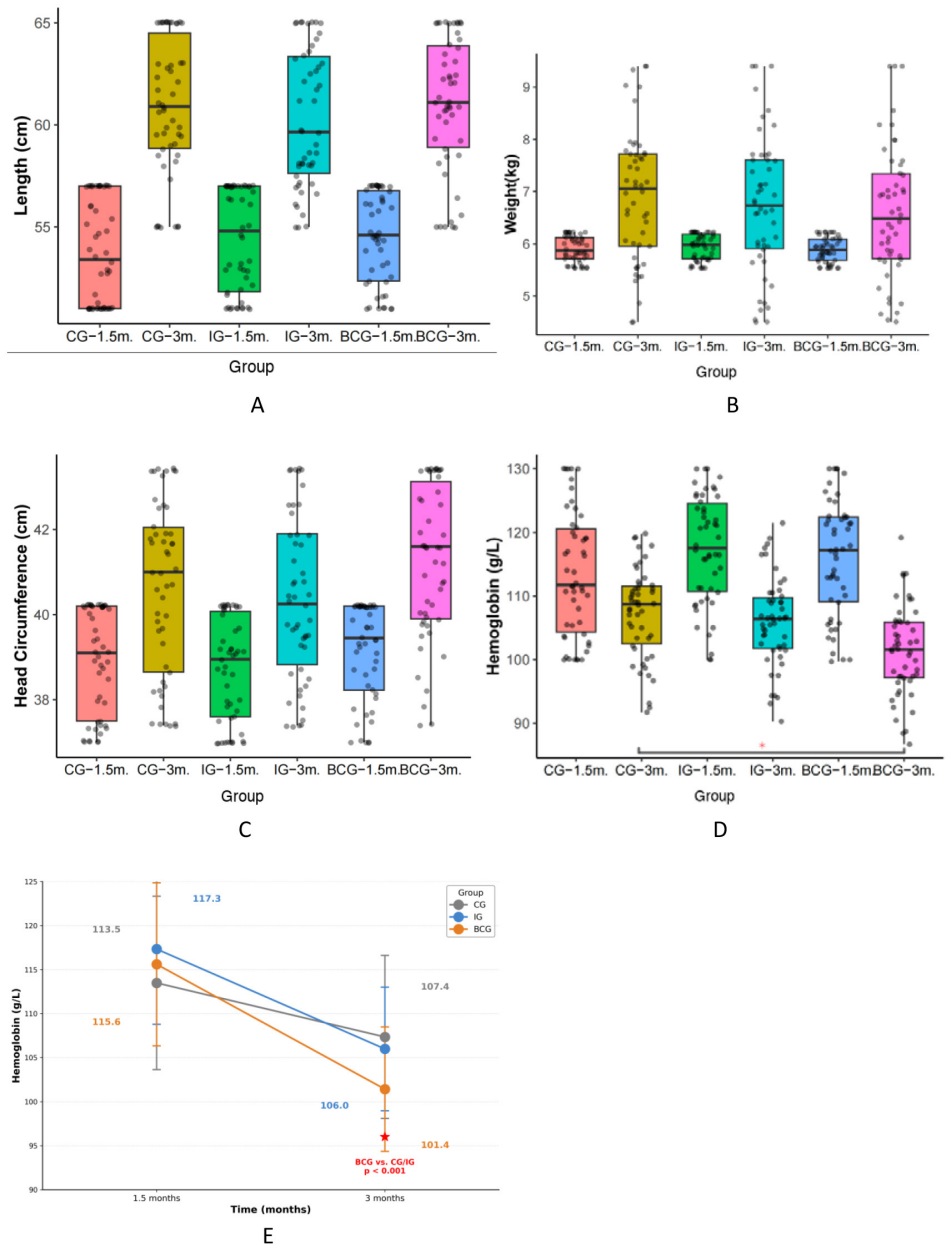
Effects of intervention on growth parameters and Hb levels. **(A)** Length; **(B)** Weight; **(C)** Head circumference; **(D)** hemoglobin; **(E)** Longitudinal changes of hemoglobin levelsCG/IG/BCG-1.5m, control group/intervention group/ breastfeeding control group at 1.5 months after birth; CG/IG/BCG-3m, control group/intervention group/ breastfeeding control group at 3 months after birth. *, significant difference between two groups.

No significant differences in weight, length, or HC were observed among the three groups at 1.5 or 3 months after birth (*p* > 0.05; Table 4, Figures 3A–C). Similarly, no statistically significant difference in Hb levels were detected among groups at 1.5 months of age (*p* > 0.05). However, at 3 months, Hb levels differed significantly among the groups (*p* < 0.05), with infants in the BCG exhibiting significantly lower Hb levels than those in both formula-fed groups (*p* < 0.05). No statistically significant difference in Hb levels was observed between the IG and CG (*p* > 0.05) (Table 4, Figures 3D, E).

**Table 4:** Growth parameters and hemoglobin level in infants during the study period (mean ± SD).

Index	CG (*N* = 50)	IG (*N* = 50)	BCG (*N* = 50)	*F* values	*p* values
Length (cm)					
1.5-month after birth^*^	53.743 ± 2.552a	54.393 ± 2.511a	54.482 ± 2.231a	1.397	0.251
3-month after birth^*^	60.890 ± 3.364a	60.274 ± 3.360a	61.021 ± 3.304a	0.719	0.489
Weight (kg)					
1.5-month after birth^*^	5.901 ± 0.254a	5.930 ± 0.254a	5.884 ± 0.230a	0.488	0.615
3-month after birth^*^	6.920 ± 1.280a	6.741 ± 1.323a	6.593 ± 1.271a	0.813	0.446
Head circumference(cm)					
1.5-month after birth^*^	38.911 ± 1.253a	38.712 ± 1.221a	39.152 ± 1.150a	1.718	0.183
3-month after birth^*^	40.630 ± 2.032a	40.360 ± 1.922a	41.244 ± 1.812a	2.827	0.062
Hemoglobin (g/L)					
1.5-month after birth^*^	113.484 ± 9.850a	117.330 ± 8.542a	115.601 ± 9.264a	2.267	0.107
3-month after birth^*^	107.35 ± 9.26a	105.990 ± 7.014a	101.420 ± 7.073b	9.629	<0.001

^*^, Student-Newman-Keuls test among the three groups; identical letters indicate no statistically significant differences among groups, while different letters indicate a statistically significant difference among groups; SD, standard deviation; IG, intervention group; CG, control group; BCG, breastfeeding control group (matched 1:1 with neonates in the IG by sex).

### 3.6 Occurrence of formula-related adverse reactions during the study period

Throughout the study period, no formula-related adverse reactions, such as abdominal cramps, nausea, vomiting, fever, diarrhea, constipation, changes in appetite, or allergy-related symptoms, were observed in the infants.

## 4 Discussion

The use of infant formula fortified with high concentrations of bioactive components – LF, MFGM, probiotics, and nucleotides–was based on the established immunological vulnerabilities of neonates delivered by C-section (25). Standard infant formulas, which historically lacked key human milk components, often failed to replicate the immunomodulatory functions necessary to compensate for C-section-associated deficits. The rationale for high-level fortification is to bridge this gap. Research and Development (R&D) initiatives have increasingly focused on developing specialized infant formulas, especially those enriched with physiological lipids (such as MFGM), specific proteins (such as LF), and selected probiotics to promote healthy gut colonization and immune development in this population, thereby reducing short- and long-term health risks (12, 26).

### 4.1 Effects of intervention on the symptoms and incidence of respiratory diseases in neonates delivered by C-section

Substantial clinical evidence supports the protective role of LF against respiratory tract infections (RTIs). Meta-analyses have shown that LF supplementation is associated with a reduced overall risk of RTIs (27, 28), and bovine lactoferrin (bLF) has been reported to significantly lower RTI incidence compared with standard formula or placebo (29). Dose-response studies further support the importance of higher LF concentrations. While bovine milk contains relatively low LF levels (0.02–0.5 g/L) (30), formulas fortified with higher bLF concentrations (e.g., 76 mg/100 g) have demonstrated greater reductions in diarrhea and RTIs compared with lower-dose formulations (31). In this study, infants receiving higher-dose LF (200 mg/100 g) exhibited fewer common disease-related symptoms than those receiving lower-dose LF (45–50 mg/100 g), suggesting a potential dose-dependent effect.

Clinical trials have also shown that supplementation with bovine MFGM (bMFGM) provides protection against infectious diseases, including reduced risk of acute otitis media (AOM), lower use of antipyretics (32), and fewer episodes of URTIs and cough (33). Similarly, probiotics, particularly when administered at sufficiently high doses, address the known deficiency of beneficial gut bacteria in C-section-delivered infants. Meta-analyses indicate that probiotics can reduce the incidence of RTIs and may also lessen symptom severity and duration (34–36), depending on strain selection and dosage (35, 37).

In contrast, nucleotide supplementation alone has not been shown to substantially reduce URTI risk (RR: 1.11, 95% CI 0.90–1.36) (38), but they may play a supportive role. Importantly, evidence increasingly suggests the synergistic benefits of multi-component fortification. Studies of formulas supplemented with both bMFGM and bLF have reported fewer respiratory adverse events and episodes of diarrhea up to 18 months of age in healthy term infants (39).

Our findings are partially consistent with the literature. Infants in the IG receiving high-dose bioactive components experienced significantly fewer total days of respiratory-related symptoms, such as coughing, runny nose, and retching/vomiting, compared with infants in the CG receiving lower-dose formulations. However, no significant differences were observed between the two groups in the incidence of bronchopneumonia, acute tracheal/bronchitis, or URTIs. Notably, respiratory symptoms and RTI incidence in the IG were numerically similar to those observed in the BCG, whereas outcomes in the CG were slightly less favorable. These results suggest that respiratory outcomes observed in the intervention group were generally similar to those observed in the breastfeeding reference group. However, the study was not designed to assess equivalence or non-inferiority relative to breastfeeding, and such conclusions cannot be drawn from the present data.

The lack of significant differences in RTI incidence between the IG and CG may be explained by several factors. First, participant heterogeneity may have reduced statistical power despite sample size estimation based on prior studies. Second, the relatively short intervention period may have been insufficient to capture long-term protective effects against RTIs. Third, although this study focused on four bioactive components, differences in other formula constituents could not be fully controlled and may have introduced bias.

### 4.2 Effects of intervention on symptoms and incidence of gastrointestinal diseases in neonates delivered by C-section

Previous randomized controlled trials (RCTs) have demonstrated that supplementation with specific strains, such as *Bifidobacterium animalis* subsp. *lactis* CNCM I-3446 or multi-strain mixtures (including *Bifidobacterium* and *Lactobacillus* genera), exerts a strong bifidogenic effect in C-section neonates. These interventions, often administered at dosages ranging from 10^7^ to 10^11^ CFU/day, have been shown to restore the gut microbiota of C-section infants to a state similar to that of vaginally delivered infants, especially when combined with prebiotics as synbiotics. However, the probiotic dosages evaluated across studies vary widely, ranging from 2 × 10^6^ to 9 × 10^11^ CFU per day (40). The concept of “high-dose” must be interpreted in relation to strain viability, delivery matrix, and host context. Current evidence remains insufficient to define optimal strains, dosages, or timing for all conditions. Some studies (41) have raised concerns that high-dose supplementation may result in only transient colonization, potentially disrupting later microbial ecology. Furthermore, contradictory findings have been reported: one study (7) showed that lower-dose probiotic supplementation produced more pronounced changes in key microbial taxa and function than higher doses. These findings suggest that probiotic efficacy is strain- and niche-specific and depends on optimal ecological saturation rather than dose alone.

In the present study, the IG formula contained 2 × 10^9^ CFU/100 g probiotics (a triple strain of *Bifidobacterium animalis* subsp. *lactis* BB-12, HN019, and Bi-07). At this dosage, combined with other bioactive nutrients in the formula, probiotic supplementation was associated with favorable trends in several gastrointestinal outcomes; however, because these analyses were secondary and the study was not powered for these endpoints, the findings should be considered exploratory. These findings suggest that the selected probiotic strains and dosages may represent a promising formulation for further investigation. However, whether this dosage is optimal for disease prevention remains unclear. Further dose-response studies are warranted to determine the optimal combination dosage of these strains.

Clinical meta-analyses have also confirmed that MFGM-supplemented formulas are safe and well-tolerated, with no significant adverse effects reported. Growth parameters were non-inferior to those of standard formula and, in some cases, more closely resemble those of breastfed infants (42), along with a lower incidence of AOM (42). The clinical efficacy of bLF appears to be context-dependent. In high-risk populations (e.g., low birth weight infants or those from low-income family), LF supplementation has been associated with reduced incidence of late-onset sepsis (43), necrotizing enterocolitis (14), infection-associated outcomes, and shorter durations of diarrhea (29). In contrast, a double-blind controlled trial (44) conducted in a low-risk population found no significant effect of bLF-fortified formula (1.0 g/L) on infection-related morbidity.

Consistent with findings for respiratory outcomes, infants in the IG experienced significantly fewer total days of gastrointestinal symptoms, including retching/vomiting, stool with milk flaps/food residues/sour odor, and refusal to eat, as well as a reduced risk of functional dyspepsia, compared with infants in the CG. The lack of statistically significant intergroup differences in other gastrointestinal symptoms and diseases may be attributed to factors similar to those discussed for respiratory outcomes. The breastfeeding group was included to provide clinical context rather than to serve as a formal comparator. Therefore, similarities between the intervention and breastfeeding groups should be interpreted descriptively rather than as evidence of equivalent efficacy.

### 4.3 Effects of intervention on symptoms and incidence of eczema in neonates delivered by C-section

C-section delivery is associated with gut microbial dysbiosis and delayed gut maturation compared with vaginal delivery, conditions that may contribute to an increased risk of immunological disorders, including atopic dermatitis (AD) or eczema (45). Preventative effects against atopic outcomes are known to be highly strain-specific and dependent on the timing and duration of intervention (46).

Fortification with MFGM has generally been shown to be safe and well-tolerated in large-scale studies, with no overall differences in skin-related adverse events or eczema incidence (47). However, a smaller, non-inferiority trial (48) highlighted the importance of MFGM processing methods. This trial compared formulas enriched with a lipid-rich fraction (MFGM-L) and a protein-rich fraction (MFGM-P). While the MFGM-L group showed a low eczema incidence (1.4%), comparable to the control group (3.5%), *post-hoc* analysis revealed a significantly higher eczema incidence (13.9%) in the MFGM-P group compared with the control (*P* = 0.01). These findings suggest that highly concentrated or structurally altered protein components in MFGM may increase immunogenicity and trigger allergic responses in susceptible infants.

Although LF is frequently included in systematic reviews evaluating strategies to reduce infant morbidity and mortality, high-quality randomized controlled trials assessing its preventive effects on eczema in high-risk infants are limited. Existing evidence indicates that LF may improve established AD through its systemic anti-inflammatory properties, suggesting a supportive rather than primary preventive role (49).

Regarding nucleotides, recent evidence has shown no association between nucleoside exposure and the occurrence of atopic outcomes, including eczema, in children under 2 years of age (50).

Although the incidence of eczema was lower in the intervention group, this outcome was a secondary endpoint and should be interpreted cautiously. The finding may represent a preliminary signal of benefit that requires confirmation in larger randomized controlled trials specifically designed to assess allergic outcomes. These findings may reflect the combined effects of LF, MFGM, probiotics, and nucleotides on intestinal flora modulation and immune regulation.

### 4.4 Effects of intervention on growth parameters and hemoglobin levels in neonates delivered by C-section

Although the microbial and immunological benefits of probiotic and synbiotic supplementation are well-documented, their direct impact on anthropometric growth outcomes in C-section-delivered infants appears largely neutral. Two studies examining the effects of probiotic (*n* = 164) and synbiotic (*n* = 193) supplementation during the 1st year of life reported no significant differences in growth parameters between the intervention and control groups (51). Similarly, infant formulas enriched with MFGM (52) generally showed no clear advantages in short-term physical growth outcomes compared with standard formulas.

Consistent findings have also been reported in cohort studies evaluating LF supplementation. One such study observed no significant differences in growth parameters among infants receiving different formulas (53), reinforcing the view that LF primarily supports immune function and metabolic regulation, such as iron homeostasis, rather than promoting accelerated physical growth in healthy infants.

Clinical evidence further indicates that nucleotide supplementation does not induce accelerated physical growth or neurological development in healthy term infants (54). However, a randomized controlled trial (54) demonstrated improved catch-up growth in term infants with severe intrauterine growth retardation when nucleotides were included in the formula, indicating that the growth-promoting effects of nucleotides are highly context-dependent.

In line with these findings, the present study showed that fortification with high-dose bioactive components had no significant effect on growth indicators in C-section-delivered neonates, regardless of dosage. This may partly reflect the limited sample size and, consequently, insufficient statistical power to detect small differences. More importantly, these results suggest that standard infant formulas already provided adequate energy and macronutrients to support normal growth. Thus, the primary roles of probiotic, MFGM, LF, and nucleotides are not to accelerate growth but to enhance safety and consistency by supporting overall health.

In contrast, the effects of intervention on Hb levels were more readily interpretable. Infant formulas supplemented with iron and LF, regardless of LF dose, provide improved iron availability, enhance iron absorption and utilization, and maintain higher Hb levels compared with exclusive breastfeeding. However, increasing LF dosage did not result in additional Hb elevation compared with lower-dose LF supplementation, likely due to the identical iron content in the formula.

### 4.5 Long-term implications and generalizability

The present study primarily provides evidence regarding the short-term safety and feasibility of a formula enriched with higher levels of probiotics, lactoferrin, MFGM, and nucleotides in term infants delivered by cesarean section. Although exploratory analyses suggested potential benefits for selected gastrointestinal and allergic outcomes, the study was not designed to establish definitive clinical efficacy for these outcomes.

Furthermore, the intervention period was limited to 3 months, and therefore the long-term health implications of this nutritional strategy remain unknown. Whether the observed findings persist beyond infancy or translate into meaningful reductions in later childhood morbidity requires further investigation.

Importantly, the present findings do not establish that the specific bioactive components included in the formula, or the dosages used, are necessary or optimal for routine clinical practice. Additional dose-response studies and larger randomized controlled trials are needed to determine the relative contribution of each component and to identify the most effective formulation.

Finally, breastfeeding remains the preferred feeding strategy whenever feasible because of its well-established nutritional, immunological, developmental, and long-term health benefits. The breastfeeding group in this study was included solely as a contextual reference and should not be interpreted as evidence that the intervention formula achieves equivalent clinical effectiveness to breastfeeding.

## 5 Limitation analysis

This study has several limitations. First, the sample size was calculated based on the primary outcome of URTIs. Therefore, the statistical power may have been insufficient to detect differences in secondary outcomes, potentially masking true intervention effects. In addition, multiple secondary outcomes were evaluated without formal adjustment for multiplicity. Consequently, the statistically significant findings observed for functional dyspepsia and eczema should be interpreted with caution, as the possibility of type I error (chance findings) cannot be excluded. Second, the bioactive component levels examined in this study represented “higher” concentrations within the national standard range rather than the “maximum” achievable doses, limiting the ability to assess dose-response relationships and optimal clinical efficacy. Third, because the intervention formula contained four bioactive components at higher levels, it was not possible to isolate the individual contribution of each component; thus, the observed effects should be interpreted as the result of the combined formulation rather than specific ingredients. Furthermore, the breastfeeding group was not randomized and was included primarily as a contextual reference. Therefore, comparisons between formula-fed and breastfed infants should be interpreted cautiously, and causal inferences regarding relative effectiveness cannot be made. Lastly, the intervention and follow-up periods were limited to 3 months. Consequently, the study was unable to evaluate whether the observed effects persist beyond early infancy or influence longer-term outcomes related to infection, allergic disease, growth, or neurodevelopment. Long-term follow-up studies are warranted.

## 6 Conclusion

In conclusion, this study found no safety concerns were identified during the 3-month supplementation period of a high-dose probiotic-LF-MFGM-nucleotide formula, supporting its safety for term neonates delivered by C-section. Although the primary outcome (URTI incidence) was not significantly different between groups, exploratory analyses suggested potential reductions in functional dyspepsia and eczema among infants receiving the high-dose bioactive formula. These findings should be considered hypothesis-generating and require confirmation in larger randomized controlled trials. The formula was safe and well tolerated in term neonates delivered by cesarean section. Breastfeeding remains the preferred standard for infant feeding whenever feasible. In the present study, the breastfeeding group served as a contextual reference, and the findings should not be interpreted as evidence that the intervention formula achieves equivalent clinical effectiveness. Larger randomized studies are needed to further evaluate the potential benefits of this formulation.

## Data Availability

The original contributions presented in the study are included in the article/supplementary material, further inquiries can be directed to the corresponding author.

## References

[1] Perin J Mulick A Yeung D Villavicencio F Lopez G Strong KL . Global, regional, and national causes of under-5 mortality in 2000–19: an updated systematic analysis with implications for the Sustainable Development Goals. Lancet Child Adolesc Health. (2022) 6:106–115. doi: 10.1016/S2352-4642(21)00311-4 34800370 PMC8786667

[2] Wang S Yin P Yu L Tian F Chen W Zhai Q. Effects of early diet on the prevalence of allergic disease in children: a systematic review and meta-analysis. Adv Nutr. (2024) 15:100128. doi: 10.1016/j.advnut.2023.10.001 37827490 PMC10831899

[3] Platts-Mills TA. The allergy epidemics: 1870–2010. J Allergy Clin Immunol. (2015) 136:3–13. doi: 10.1016/j.jaci.2015.03.048 26145982 PMC4617537

[4] Wong GWK Li J Bao YX Wang JY Leung TF Li LL . Pediatric allergy and immunology in China. Pediatr Allergy Immunol. (2018) 29:127–32. doi: 10.1111/pai.12819 29047174

[5] Amabebe E Anumba DOC. The vaginal microenvironment: the physiologic role of *lactobacilli*. Front Med. (2018) 5:181. doi: 10.3389/fmed.2018.00181 PMC6008313 29951482

[6] Cukrowska B Bierła JB Zakrzewska M Klukowski M Maciorkowska E. The relationship between the infant gut microbiota and allergy. The role of *Bifidobacterium breve* and prebiotic oligosaccharides in the activation of anti-allergic mechanisms in early life. Nutrients. (2020) 12:946. doi: 10.3390/nu12040946 32235348 PMC7230322

[7] Inchingolo F Inchingolo AD Palumbo I Trilli I Guglielmo M Mancini A . The impact of cesarean section delivery on intestinal microbiota: mechanisms, consequences, and perspectives—A systematic review. Int J Mol Sci. (2024) 25:1055. doi: 10.3390/ijms25021055 38256127 PMC10816971

[8] Gomaa EZ. Human gut microbiota/microbiome in health and diseases: a review. Antonie Van Leeuwenhoek. (2020) 113:2019–40. doi: 10.1007/s10482-020-01474-7 33136284

[9] Chandrasekaran P Weiskirchen S Weiskirchen R. Effects of probiotics on gut microbiota: an overview. Int J Mol Sci. (2024) 25:6022. doi: 10.3390/ijms25116022 38892208 PMC11172883

[10] Tan GSE Tay HL Tan SH Lee TH Ng TM Lye DC. Gut microbiota modulation: implications for infection control and antimicrobial stewardship. Adv Ther. (2020) 37:4054–67. doi: 10.1007/s12325-020-01458-z 32767183 PMC7412295

[11] Milani C Duranti S Bottacini F Casey E Turroni F Mahony J . The first microbial colonizers of the human gut: composition, activities, and health implications of the infant gut microbiota. Microbiol Mol Biol Rev. (2017) 81:e00036–17. doi: 10.1128/MMBR.00036-17 PMC5706746 29118049

[12] Chong HY Tan LT Law JW Hong KW Ratnasingam V Ab Mutalib NS . Exploring the potential of human milk and formula milk on infants' gut and health. Nutrients. (2022) 14:3554. doi: 10.3390/nu14173554 36079814 PMC9460722

[13] Moubareck CA. Human milk microbiota and oligosaccharides: a glimpse into benefits, diversity, and correlations. Nutrients. (2021) 13:1123. doi: 10.3390/nu13041123 33805503 PMC8067037

[14] Carr LE Virmani MD Rosa F Munblit D Matazel KS Elolimy AA . Role of human milk bioactives on infants' gut and immune health. Front Immunol. (2021) 12:604080. doi: 10.3389/fimmu.2021.604080 33643310 PMC7909314

[15] Moossavi S Miliku K Sepehri S Khafipour E Azad MB. The prebiotic and probiotic properties of human milk: implications for infant immune development and pediatric asthma. Front Pediatr. (2018) 6:197. doi: 10.3389/fped.2018.00197 30140664 PMC6095009

[16] Conesa C Bellés A Grasa L Sánchez L. The role of lactoferrin in intestinal health. Pharmaceutics. (2023) 15:1569. doi: 10.3390/pharmaceutics15061569 37376017 PMC10304194

[17] Yao D Ranadheera CS Shen C Wei W Cheong LZ. Milk fat globule membrane: composition, production and its potential as encapsulant for bioactives and probiotics. Crit Rev Food Sci Nutr. (2024) 64:12336–51. doi: 10.1080/10408398.2023.2249992 37632418

[18] Doo EH Chassard C Schwab C Lacroix C. Effect of dietary nucleosides and yeast extracts on composition and metabolic activity of infant gut microbiota in PolyFermS colonic fermentation models. FEMS Microbiol Ecol. (2017) 93. doi: 10.1093/femsec/fix088 28854667

[19] Singhal A Macfarlane G Macfarlane S Lanigan J Kennedy K Elias-Jones A . Dietary nucleotides and fecal microbiota in formula-fed infants: a randomized controlled trial. Am J Clin Nutr. (2008) 87:1785–92. doi: 10.1093/ajcn/87.6.1785 18541569

[20] Cai J Li S Wei Q Wang H Jiang Y Jiao J. Nutritional composition dataset of approved infant formula powder in China (2017–2024). Data Brief . (2025) 61:111895. doi: 10.1016/j.dib.2025.111895 40756425 PMC12318258

[21] Miliku K Azad MB. Breastfeeding and the developmental origins of asthma: current evidence, possible mechanisms, and future research priorities. Nutrients. (2018) 10:995. doi: 10.3390/nu10080995 30061501 PMC6115903

[22] Zhonghua Medical Association Zhonghua Medical Association Publishing House General General Practice Branch of Zhonghua Medical Association . Guidelines for the diagnosis and treatment of acute upper respiratory tract infections in primary care (practical edition 2018). Chin J Gen Pract. (2019) 18:427–30. doi: 10.3760/cma.j.issn.1671-7368.2019.05.006

[23] Shah VB Shah BS Puranik GV. Evaluation of non cyanide methods for hemoglobin estimation. Indian J Pathol Microbiol. (2011) 54:764–8. doi: 10.4103/0377-4929.91494 22234106

[24] Kara SS Volkan B Erten I. Lactobacillus rhamnosus GG can protect malnourished children. Benef Microbes. (2019) 10:237–44. doi: 10.3920/BM2018.0071 30638398

[25] Madan JC Hoen AG Lundgren SN Farzan SF Cottingham KL Morrison HG . Association of cesarean delivery and formula supplementation with the intestinal microbiome of 6-week-old infants. JAMA Pediatr. (2016) 170:212–9. doi: 10.1001/jamapediatrics.2015.3732 26752321 PMC4783194

[26] Ahern GJ Hennessy AA Ryan CA Ross RP Stanton C. Advances in infant formula science. Annu Rev Food Sci Technol. (2019) 10:75–102. doi: 10.1146/annurev-food-081318-104308 30908947

[27] Berthon BS Williams LM Williams EJ Wood LG. Effect of lactoferrin supplementation on inflammation, immune function, and prevention of respiratory tract infections in humans: a systematic review and meta-analysis. Adv Nutr. (2022) 13:1799–819. doi: 10.1093/advances/nmac047 35481594 PMC9526865

[28] Ali AS Hasan SS Kow CS Merchant HA. Lactoferrin reduces the risk of respiratory tract infections: a meta-analysis of randomized controlled trials. Clin Nutr ESPEN. (2021) 45:26–32. doi: 10.1016/j.clnesp.2021.08.019 34620326

[29] Mayorga V Navarro R Roldan V Urtecho M Tipe S Calvert B . Efficacy of lactoferrin supplementation in pediatric infections: a systematic review and meta-analysis. Biochem Cell Biol. (2025) 103:1–23. doi: 10.1139/bcb-2024-0181 39841980

[30] Bakshi S Paswan VK Yadav SP Bhinchhar BK Kharkwal S Rose H . A comprehensive review on infant formula: nutritional and functional constituents, recent trends in processing and its impact on infants' gut microbiota. Front Nutr. (2023) 10:1194679. doi: 10.3389/fnut.2023.1194679 37415910 PMC10320619

[31] Chen K Jin S Chen H Cao Y Dong X Li H . Dose effect of bovine lactoferrin fortification on diarrhea and respiratory tract infections in weaned infants with anemia: a randomized, controlled trial. Nutrition. (2021) 90:111288. doi: 10.1016/j.nut.2021.111288 34102559

[32] Timby N Hernell O Vaarala O Melin M Lönnerdal B Domellöf M. Infections in infants fed formula supplemented with bovine milk fat globule membranes. J Pediatr Gastroenterol Nutr. (2015) 60:384–9. doi: 10.1097/MPG.0000000000000624 25714582

[33] Mohamed HJJ Lee EKH Woo KCK Sarvananthan R Lee YY Mohd Hussin ZA. Brain-immune-gut benefits with early life supplementation of milk fat globule membrane. JGH Open. (2022) 6:454–61. doi: 10.1002/jgh3.12775 35822117 PMC9260205

[34] Zhao Y Dong BR Hao Q. Probiotics for preventing acute upper respiratory tract infections. Cochrane Database Syst Rev. (2022) 8:CD006895. doi: 10.1002/14651858.CD006895.pub4 36001877 PMC9400717

[35] Zhang Y Xu Y Hu L Wang X. Advancements related to probiotics for preventing and treating recurrent respiratory tract infections in children. Front Pediatr. (2025) 13:1508613. doi: 10.3389/fped.2025.1508613 39981209 PMC11839809

[36] Vouloumanou EK Makris GC Karageorgopoulos DE Falagas ME. Probiotics for the prevention of respiratory tract infections: a systematic review. Int J Antimicrob Agents. (2009) 34:197.e1–197.e10. doi: 10.1016/j.ijantimicag.2008.11.005 19179052

[37] Depoorter L Vandenplas Y. Probiotics in pediatrics. A review and practical guide. Nutrients. (2021) 13:2176. doi: 10.3390/nu13072176 34202742 PMC8308463

[38] Gutiérrez-Castrellón P Mora-Magaña I Díaz-García L Jiménez-Gutiérrez C Ramirez-Mayans J Solomon-Santibáñez GA. Immune response to nucleotide- supplemented infant formulae: systematic review and meta-analysis. Br J Nutr. (2007) 98:S64–7. doi: 10.1017/S000711450783296X 17922963

[39] Chichlowski M Bokulich N Harris CL Wampler JL Li F Berseth CL . Effect of bovine milk fat globule membrane and lactoferrin in infant formula on gut microbiome and metabolome at 4 months of age. Curr Dev Nutr. (2021) 5:nzab027. doi: 10.1093/cdn/nzab027 33981943 PMC8105244

[40] Martín-Peláez S Cano-Ibáñez N Pinto-Gallardo M Amezcua-Prieto C. The impact of probiotics, prebiotics, and synbiotics during pregnancy or lactation on the intestinal microbiota of children born by cesarean section: a systematic review. Nutrients. (2022) 14:341. doi: 10.3390/nu14020341 35057522 PMC8778982

[41] Quin C Estaki M Vollman DM Barnett JA Gill SK Gibson DL. Probiotic supplementation and associated infant gut microbiome and health: a cautionary retrospective clinical comparison. Sci Rep. (2018) 8:8283. doi: 10.1038/s41598-018-26423-3 29844409 PMC5974413

[42] Ambrożej D Dumycz K Dziechciarz P Ruszczyński M. Milk fat globule membrane supplementation in children: systematic review with meta-analysis. Nutrients. (2021) 13:714. doi: 10.3390/nu13030714 33668227 PMC7996302

[43] Ochoa TJ Zegarra J Cam L Llanos R Pezo A Cruz K . Randomized controlled trial of lactoferrin for prevention of sepsis in Peruvian neonates less than 2500 g. Pediatr Infect Dis J. (2015) 34:571–576. doi: 10.1097/INF.0000000000000593 25973934 PMC4435832

[44] Björmsjö M Hernell O Lönnerdal B Berglund SK. Immunological effects of adding bovine lactoferrin and reducing iron in infant formula: a randomized controlled trial. J Pediatr Gastroenterol Nutr. (2022) 74:e65–72. doi: 10.1097/MPG.0000000000003367 34908015 PMC8860203

[45] Long G Hu Y Tao E Chen B Shu X Zheng W . The influence of cesarean section on the composition and development of gut microbiota during the first 3 months of life. Front Microbiol. (2021) 12:691312. doi: 10.3389/fmicb.2021.691312 34489887 PMC8416498

[46] Chu DK Koplin JJ Ahmed T Islam N Chang CL Lowe AJ. How to prevent atopic dermatitis (Eczema) in 2024: theory and evidence. J Allergy Clin Immunol Pract. (2024) 12:1695–704. doi: 10.1016/j.jaip.2024.04.048 38703820

[47] Fontecha J Brink L Wu S Pouliot Y Visioli F Jiménez-Flores R. Sources, production, and clinical treatments of milk fat globule membrane for infant nutrition and well-being. Nutrients. (2020) 12:1607. doi: 10.3390/nu12061607 32486129 PMC7352329

[48] Billeaud C Puccio G Saliba E Guillois B Vaysse C Pecquet S . Safety and tolerance evaluation of milk fat globule membrane-enriched infant formulas: a randomized controlled multicenter non-inferiority trial in healthy term infants. Clin Med Insights Pediatr. (2014) 8:51–60. doi: 10.4137/CMPed.S16962 25452707 PMC4219856

[49] Presti S Manti S Parisi GF Papale M Barbagallo IA Li Volti G . Lactoferrin: cytokine modulation and application in clinical practice. J Clin Med. (2021) 10:5482. doi: 10.3390/jcm10235482 34884183 PMC8658270

[50] Timmermans MJ Dagnelie PC Theunisz EH Ewalds D Thijs C Mommers M . Dietary nucleotide and nucleoside exposure in infancy and atopic dermatitis, recurrent wheeze, and allergic sensitization. J Pediatr Gastroenterol Nutr. (2015) 60:691–3. doi: 10.1097/MPG.0000000000000689 25564817

[51] Kamphorst K Carpay NC de Meij TGJ Daams JG van Elburg RM Vlieger AM. Clinical outcomes following pre-, pro- and synbiotic supplementation after caesarean birth or antibiotic exposure in the first week of life in term born infants: a systematic review of the literature. Front Pediatr. (2022) 10:974608. doi: 10.3389/fped.2022.974608 36299694 PMC9589227

[52] Li X Peng Y Li Z Christensen B Heckmann AB Stenlund H . Feeding infants formula with probiotics or milk fat globule membrane: a double-blind, randomized controlled trial. Front Pediatr. (2019) 7:347. doi: 10.3389/fped.2019.00347 31552203 PMC6736587

[53] Anticona C Esberg A Berglund SK Björmsjö M Hernell O Lönnerdal B . Impact of bovine lactoferrin supplementation and reduced iron in formula on infant oral microbiome: a randomized controlled trial. J Oral Microbiol. (2025) 17:2561212. doi: 10.1080/20002297.2025.2561212 41020047 PMC12466180

[54] Yu VY. The role of dietary nucleotides in neonatal and infant nutrition. Singapore Med J. (1998) 39:145–50. 9676143

